# Application of PLC and HMI in the CO_2_ transcritical refrigeration experimental platform

**DOI:** 10.1038/s41598-022-19602-w

**Published:** 2022-09-07

**Authors:** Jianye Gao, Jinfeng Wang, Jing Xie

**Affiliations:** 1 College of Food Science and Technology, Shanghai Ocean University, Shanghai, 201306 China; 2 Shanghai Professional Technology Service Platform on Cold Chain Equipment Performance and Energy Saving Evaluation, Shanghai, 201306 China; 3 Shanghai Engineering Research Center of Aquatic Product Processing & Preservation, Shanghai, 201306 China; 4 National Experimental Teaching Demonstration Center for Food Science and Engineering, Shanghai Ocean University, Shanghai, 201306 China

**Keywords:** Energy infrastructure, Mechanical engineering

## Abstract

The paper adopted SIMATIC PLC as the control center and cooperates with SIMATIC KTP900 basic touch screen and GRM533YW-C IOT module to design a CO_2_ transcritical refrigeration experiment platform (EXP). The EXP acquired analog signals from sensors through PLC expansion modules. The PLC communicated with the touch screen and the IoT module through PROFINET to achieve data interaction. In the EXP, the touch screen and the remote devices had separate control interfaces, both of which can perform system control and real-time data display. The control strategy and abnormal alarm of CO_2_ transcritical refrigeration system was accomplished in PLC. In the cooling experiment, the maximum deviation value of temperature was less than 0.4 °C in the refrigeration container. In the 750 W load step experiment, the static error of the temperature was ± 0.2 °C in the refrigeration container, and the static error of the superheat was ± 0.17 K. This indicated that the EXP had excellent control quality. The different control strategies for the compressor, gas cooler fan, auxiliary cooler fan, EEV and pressure regulating valve can be realized in the EXP. Therefore, the performance optimization of CO_2_ transcritical refrigeration system in different operating conditions can be studied.

## Introduction

The Internet of Things (IOT) and energy conservation and emission reduction are the recent development direction of multiple industries. In the refrigeration field, due to ozone layer depletion, HFCs and Cloro-hydrofluorocarbons CHFCs will be phased out by 2040^1^. The EPA estimates that cutting the use of refrigerants in air conditioners industry alone could reduce CO_2_ emissions by 994 million equivalent tons by 2030. This accounts for 22% of the non-CO_2_ greenhouse gas reduction potential^2^. The CO_2_ as a natural refrigerant is cheap and easy to obtain, with excellent thermophysical properties, and its GWP and ODP are 1 and 0 respectively. Therefore, the use of CO_2_ as refrigerant is environmentally friendly and is one of the important initiatives to reduce carbon emissions and achieve carbon neutrality^3,4^.

Programmable Logic Controller (PLC) is often used as main controller in industrial control^5,6^. The PLC can be programmed and storage programs inside it, which can realize receiving signals from sensors and manual inputs, and controlling the actuators by digital or analog signals^7,8^. PLC has the characteristics of modularity, expandability and high reliability. And Siemens series PLC supports a variety of Human Machine Interaction operating terminals. The Human Machine Interface (HMI) can graphically display the parameters and control processes in the system. It is able to collect data from the controlled system and send control commands to the controlled system remotely and in real-time, thereby improving the efficiency of control^9–11^.

In recent years, researchers in various fields have combined PLC and HMI to design systems and to apply and analyze the designed systems. Chang et al.^12^ designed a single-tube heat transfer experiment rig and developed a measurement and control platform based on PLC and HMI, reducing the difficulty of equipment operation. Butuza et al.^13^ designed an automation solution for monitoring and controlling a micro hydropower plant based on PLC. It was used to detect real-time data and control the operation of the hydroelectric power plant. José Carlos et al.^14^ designed a potable water discharge control and monitoring system platform based on PLC and HMI. Real-time monitoring of variables and fault detection were realized to help users to solve faults quickly. Guo et al.^15^ designed an evaporative cooling simulation platform based on PLC in order to verify the feasibility of the evaporative cooling system applied in supercomputers. Pan et al.^16^ developed a monitoring platform for helium refrigerator using the configuration software Step7 of Siemens PLC and the industrial monitoring software WinCC. Although the above studies can satisfy the special functions of the system. However, the above studies mostly emphasize the application of the combination of PLC and HMI. The design of the combination of PLC and HMI was rarely mentioned. And there was no remote-control function. These systems were not combined with master computer software to achieve system control, such as LabVIEW, thus the expansion of control strategies for these systems relatively poor. In the paper, the system was controlled remotely through the IOT module, and the control strategy of the system was expanded through LabVIEW.

In order to complete an experimental study on heat transfer of gas cooler under the specific discharge pressure, Xiang Qin et al.^17^ designed the transcritical CO_2_ heat pump experimental system. This system was experimented by controlling the discharge pressure at 7920, 8010, 8120, 8520 and 8730 kPa and the compressor frequency at 20, 25, 30, 35 and 40 Hz. Anci Wang et al.^18^ established the test bench for evaluating the performance of the transcritical CO_2_ electric vehicle air conditioning system. The test bench was experimented by controlling the compressor speed at 3500, 5000 and 6500 rpm. In 17 and 18, the number of controlled devices in the system is few, and the number of target operating conditions available through the control devices is few. In the paper, the controlled devices target values were compressor frequency, gas cooler fan speed, auxiliary cooler fan speed, electronic expansion valve opening and pressure regulating valve opening, etc. The five controlled devices target values can be regulated steplessly within the adjustment range to obtain any experimentally required operating conditions. Laura Nebot-Andr´es et al.^19^ established the experimental CO_2_ plant used to obtain optimal operating conditions at gas cooler exit temperatures of 27.5 °C, 32.5 °C and 37.5 °C and evaporation levels of −15.0 °C, −10.0 °C and −5.0 °C. In 19, the devices were regulated singly. The plant cannot be optimally controlled for multiple objectives by combined control of multiple devices. In the EXP, the system was integrated online control through the HMI. The combined control of compressor frequency, gas cooler fan speed, auxiliary cooler fan speed, electronic expansion valve opening and pressure regulating valve opening was carried out to achieve multiple objectives of operation control strategy optimization: the maximum COP, the maximum exergy efficiency and the minimum carbon emission.

This paper conducts the design and realization of CO_2_ transcritical refrigeration experimental platform (EXP). The EXP enables operational control and data acquisition of CO_2_ transcritical refrigeration system. And the experimental data of EXP was collected and saved remotely and in real-time through HMI. In addition, the control system was improved. The number of acquisition points of EXP was further extended. The control methods and the ways to achieve control were further expanded. The EXP realizes different control methods of various equipment operation in the system. And the system abnormal data detection and processing were realized. The experimental study of different control strategies^20,21^ for CO_2_ transcritical refrigeration system can be carried out in the EXP.

## Principle and equipment of CO_2_ transcritical refrigeration experiment platform

**Figure 1:**
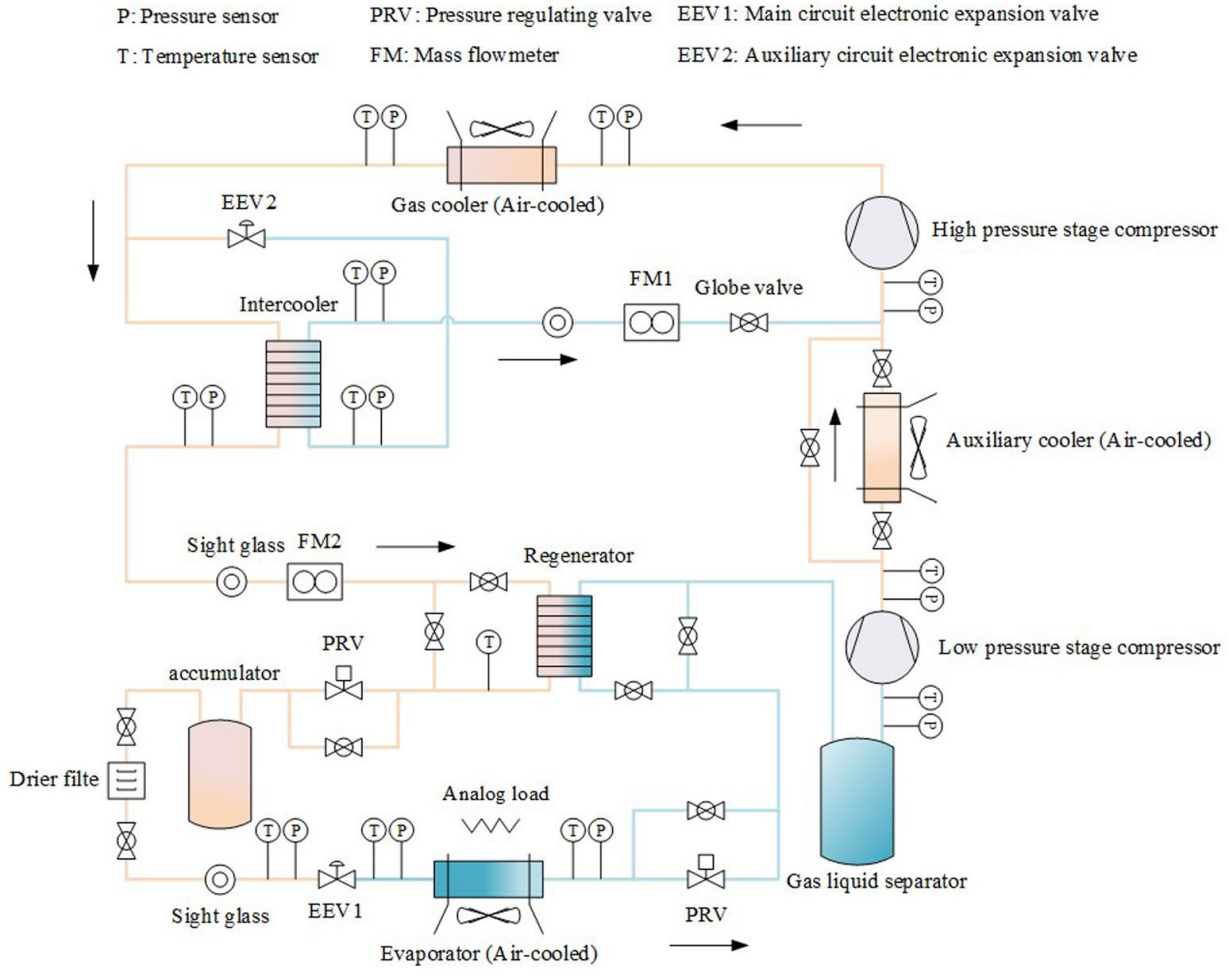
System schematic diagram of the CO_2_ transcritical refrigeration experimental rig.

The system schematic of the CO_2_ transcritical refrigeration^22^ experimental rig is shown in Fig. 1. In this system, an auxiliary cooler was added to improve the COP of the system and reduce exergy destruction of the system. A 0.2 kW heating device was added to the inside of the refrigeration container door for anti-icing. In this system, an analog load was added to simulate heat of the goods. The power of the analog load can be adjusted in the range of 0–5 kW.

In the EXP, the compressor speed can be controlled by PID controller and manually. The fan speed can regulate the heat transfer of the gas cooler and auxiliary cooler in the EXP respectively. The fan speed is controlled by PID controller and manually. The two pressure regulating valves control the discharge pressure of the gas cooler and the suction pressure of the low-pressure stage compressor, respectively. The gas cooler discharge pressure can be adjusted in real time by the optimal pressure corresponding to the gas cooler discharge temperature. The pressure regulating valve is controlled by PID controller and manually. The main circuit EEV can control the superheat of the evaporator outlet refrigerant by superheat controller, and the main circuit EEV can control the pressure of the evaporator outlet refrigerant by PID pressure controller. The EXP can realize the combined control between components with the goal of maximum COP, the maximum exergy efficiency and the minimum carbon emission or other evaluation indexes. Thus, the system energy consumption can be reduced. In the EXP, the auxiliary cooler and the regenerator can be closed by opening the bypass valve next to the auxiliary cooler or the regenerator, respectively. This enables the effect of auxiliary cooler and regenerators on the refrigeration system to be studied in the EXP.

The devices of the CO_2_ transcritical refrigeration experimental rig is shown in Table 1. Among them, the compressor adopts DC brushless motor, and the compressor speed can be adjusted in 37 ~ 100rps by inverter. The compressor is double rotor type compound compressor, the use of the compound compressor can reduce the refrigerant discharge temperature and also enhance the volumetric efficiency^23–25^. The pressure sensor in the EXP has a range of −1 to 159 bar, with an accuracy of ± 0.5%. The temperature sensor in the EXP uses platinum resistance, which is used to acquire the temperature during the refrigeration system cycle, with a range of −40 to 80 °C and an accuracy of ± 0.1 °C. The EXP also has 16 removable thermocouple temperature sensors with a range of −100 to 400 °C and an accuracy of ± 0.2 °C. The thermocouple temperature sensors can be placed according to experimental needs. The range of mass flow meter FM1 is 0–20 kg/h with an accuracy of ± 2.0%, and the range of mass flow meter FM2 is 0–100 kg/h with an accuracy of ± 2.0%. The maximum pressure of the CO_2_ transcritical refrigeration system reaches about 90 bar during operation. For safety, the stainless-steel pipes were used in the refrigeration system.

**Table 1:** The devices of the CO_2_ transcritical refrigeration experimental rig.

Name	Type	Specification
Variable speed compressor	Panasonic C-CV163L0A	Exhaust volume: 4.5 cm^3^/rev Rated cooling capacity: 3720 W
Gas cooler (Air-cooled)	Finned heat exchanger	116 mm*490 mm*430 mm Heat transfer area: 0.8682m^2^
Auxiliary cooler (Air-cooled)	Finned heat exchanger	90 mm*350 mm*330 mm Heat transfer area: 0.3969 m^2^
Evaporator (Air-cooled)	Finned heat exchanger	255 mm*595 mm*310 mm Heat transfer area: 2.0242m^2^
Intercooler	Plate type heat exchanger	73 mm*187 mm*18 mm
Regenerator	Plate type heat exchanger	73 mm*187 mm*18 mm
Gas–liquid separator	PKHQ-16-CDM	Volume: 1.3 L Maximum working pressure: 60 bar
Accumulator	PKHC-1.2/16–12-CDH	Volume: 1.2 L Maximum working pressure: 140 bar
Globe valve	PKHJ-04S/07F	Maximum working pressure: 140 bar
Drier filte	PKHE-033S-CDH	Volume: 0.08 L Maximum working pressure: 140 bar
Electronic expansion valve	UKV-J14D	Flow coefficient: 0.067 Maximum working pressure: 150 bar
Pressure regulating valve	CCMT2-027H7200	Kv value: 0.170 m^3^/h Maximum working pressure: 140 bar
Pressure sensor	AKS2050	Measuring range: −1 to 159 bar Accuracy: ± 0.5%
Temperature sensor	RTD Sensor (PT100)	Measuring range: −40 to 80℃ Accuracy: ± 0.1℃
Temperature sensor	Thermocouples	Measuring range: −100 to 400℃ Accuracy: ± 0.2℃
Mass flowmeter (FM1)	ACU20FD-MM	Measuring range: 0 to 20 kg/h Accuracy: ± 2.0%
Mass flowmeter (FM2)	DMF-1-S6	Measuring range: 0–100 kg/h Accuracy: ± 2.0%
Stainless-steel pipe	Pressure resistance of 150 bar	-

## Design of control system in CO_2_ transcritical refrigeration experimental platform

**Figure 2:**
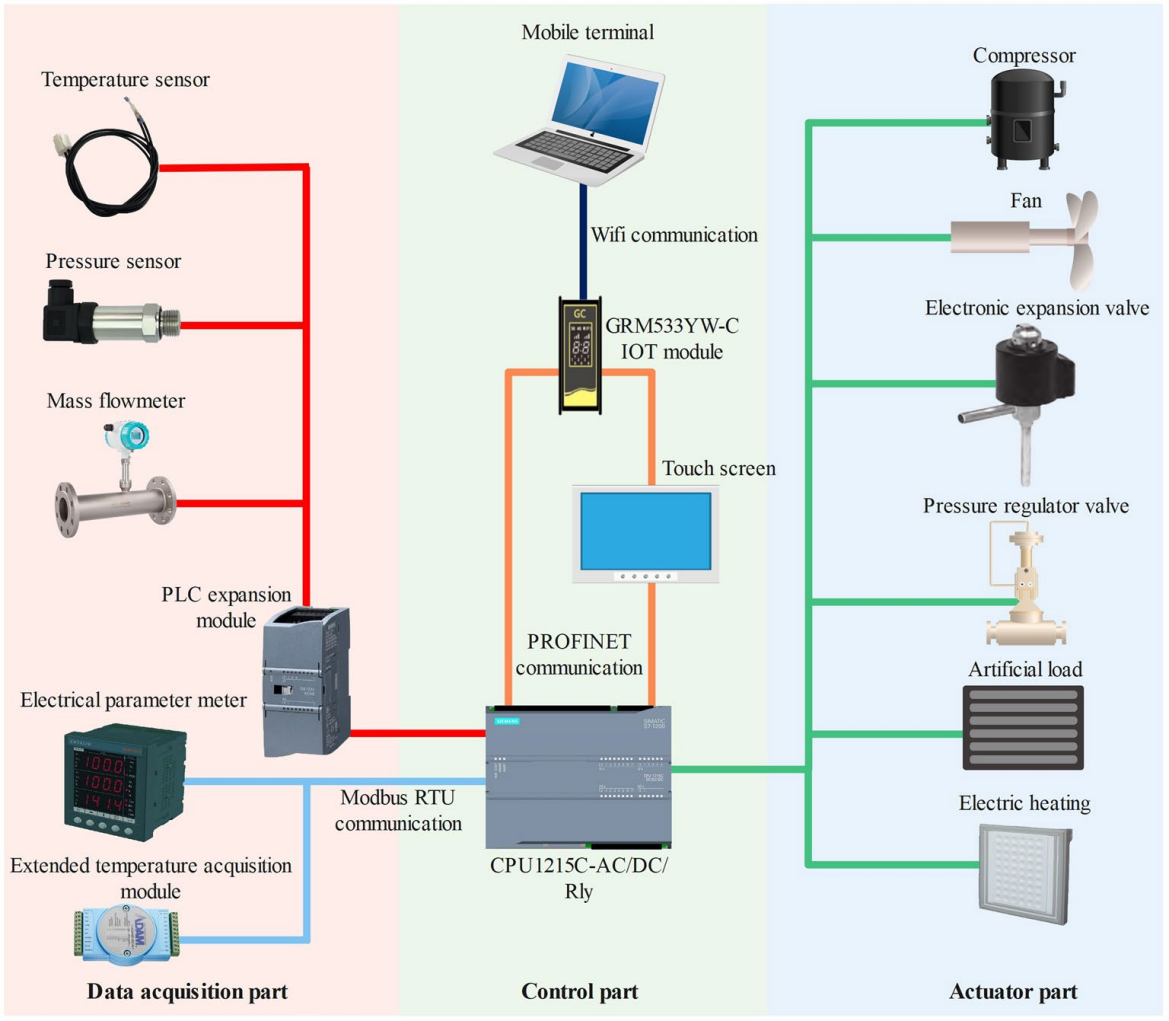
CO_2_ transcritical refrigeration experimental platform control system structure.

**Figure 3:**
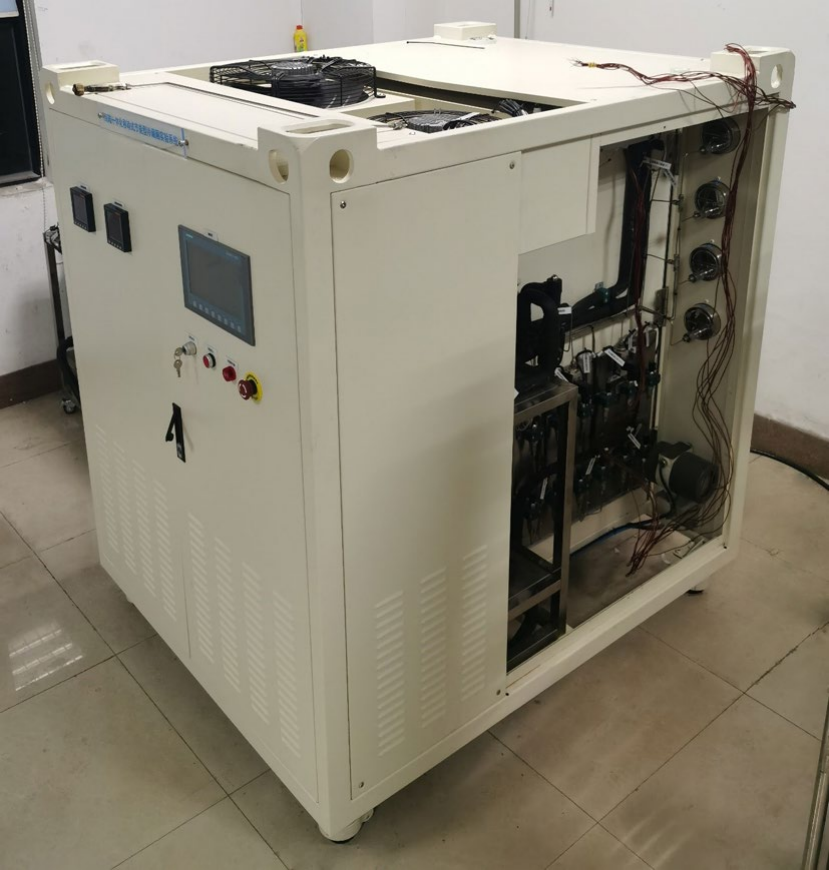
CO_2_ transcritical refrigeration experimental platform.

The controller enables stable operation of the control system^26^. In the EXP, Siemens S7-1200 series PLC was selected as the controller. The control system structure schematic diagram of the CO_2_ transcritical refrigeration experimental platform is shown in Fig. 2. The CO_2_ transcritical refrigeration experimental platform is shown in Fig. 3. The control system was composed of the data acquisition part, the control part and the actuator part. In the control part, the PLC receives signals from the data acquisition part, and the control signals processed by the PLC were sent to the actuator part. The output signals of PLC include digital and analog signals. The PLC data interaction with the touch screen through PROFINET, and researchers can send control commands and receive data from the experiment rig on the touch screen. GRM533YW-C IOT module communicates with touch screen and PLC through PROFINET. IOT module enables mobile devices to remotely monitor data from the experiment rig.

### PLC control system design

**Figure 4:**
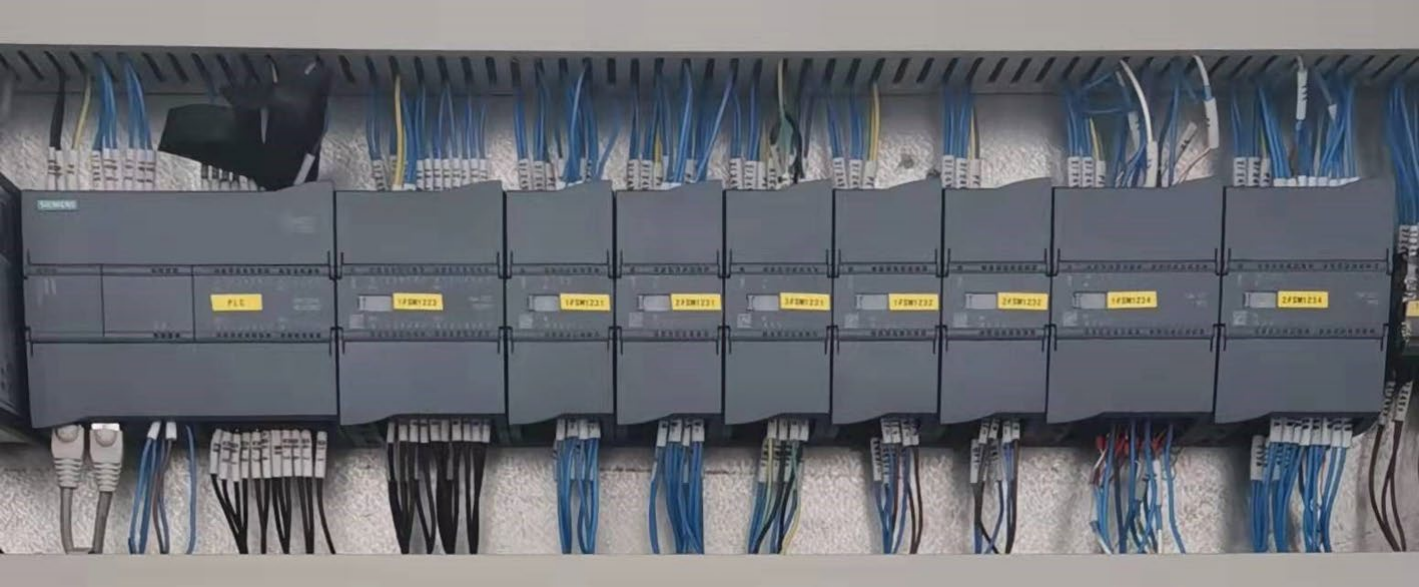
The PLC module in the EXP.

The central processing unit of the PLC S7-1200 was CPU 1215C-AC/DC/Rly, which can be expanded with eight signal modules. The PLC modules were fixed by DIN rail. In the EXP, the CPU through back plane connection to expand one digital input/output module (SM 1223 DI16/DQ16 × relay), three analog input modules (SM 1231 AI8 × 13 bit), two analog output modules (SM 1232 AQ4 × 14bit) and two thermistor analog input modules(SM 1231 AI8 × RTU). The PLC module in the EXP is shown in Fig. 4. According to the above module model, the modules were configured by TIA Portal V16 software to make the program compatible with the modules. The sensors connected with the PLC through signal lines, these PLC signal input modules were used to collect alarm, temperature, pressure and flow signals from the sensors. The alarm signals were collected by the SM 1223 DI16/DQ16 × relay module. The pressure and flow signals were collected by the SM1231 AI8 × 13bit module. The four resistance signals from the platinum resistance temperature sensors were converted to 4–20 mA current signals by the temperature transmitter (SIN-ST500) and then collected by the SM1231 AI8 × 13bit module. The sixteen resistance signals from the platinum resistance temperature sensors were collected directly by the SM1231 AI8 × RTU module. The SM1231 AI8 × 13bit module converts current signals into CPU recognizable signals in the corresponding range of 0—27,648. In the CPU, the signals in the range 0–27,648 were converted to the corresponding display data according to the range of temperature, pressure and flow, respectively. The signals collected by the AI8 × RTU module were converted to the corresponding temperature data by dividing by 10. The converted data were stored in the data register DB2.

The eight PLC expansion modules cannot meet the required number of acquisition points in the EXP. The communication board (CB 1241 RS485) was added to the PLC to further expand the number of acquisition points of the PLC. The two electrical parameter meters (EPM) and the two extended temperature acquisition modules (ETAM) were connected to the communication board to establish Modbus RTU protocol communication. The PLC was used as the master station. The two electrical parameter meters (EPM) and the two extended temperature acquisition modules (ETAM) were used as slave stations. The CPU polls the slave stations, reads the slave station data about every 10 ms, and stores the data in the PLC data register M to achieve real-time data acquisition and storage. The master and slave station communication parameters should be the same. Master station communication parameters were set by TIA Portal V16 software. The communication parameters settings of the master and slave station are shown in Table 2. The two EPM acquire the electrical parameters of the compressor and the analog load, the electrical parameters include voltage, current, power and frequency values. The two ETAM had 16 removable temperature acquisition points. The data from ETAM and EPM were stored in the PLC data register DB2.

**Table 2:** The master and slave station communication parameters setting.

Description	Set
Interface type	RS-485
Data type	Word
EPM station number	1, 2
ETAM station number	3, 4
DATA_ADDR(EPM)	44,097
DATA_ADDR(ETAM)	40,001
DATA_LEN(EPM)	10
DATA_LEN(ETAM)	8
Baud rate	9600

**Figure 5:**
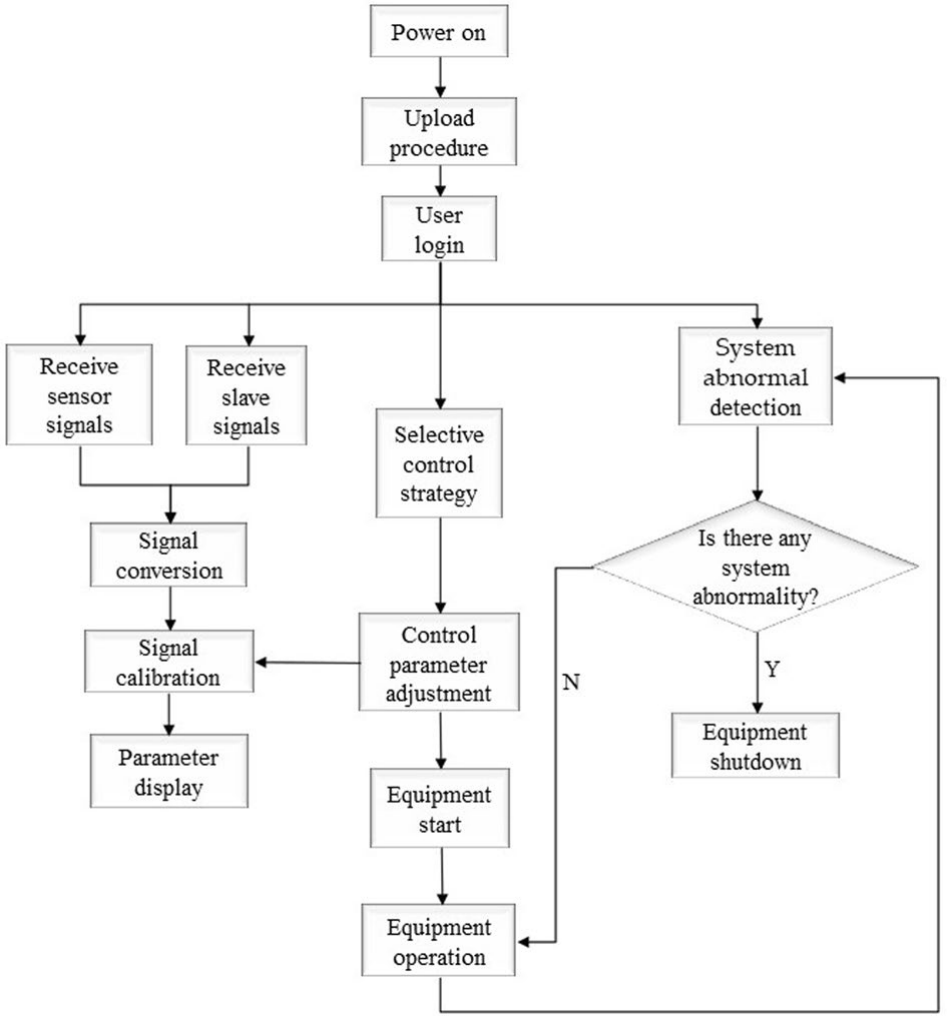
The running process of the CO_2_ transcritical refrigeration experimental platform.

The digital and analog signals were sent to the actuators by the SM 1223 DI16/DQ16 × relay module and the SM 1232 AQ4 × 14bit module. The 0–10 V DC voltage signals from the SM 1232 AQ4 × 14bit module to control the speed of the compressor and fan, the opening of the EEV and pressure regulating valve. The 4–20 mA current signal from the SM 1232 AQ4 × 14bit module controls the power of the analog load. The data conversion and logic judgment of the EXP was realized by programming in the PLC. The PLC control program was written by TIA Portal V16 software. Data conversion and calibration of signals, and equipment start/stop was achieved by programming FB blocks. The FB block was invoked by the OB block. Data conversion and calibration of different signals and several equipment start/stop in the EXP was achieved through OB block programming. The start/stop program controls the equipment including the compressor, gas cooler fan, auxiliary cooler fan, evaporator fan, analog load and door electric heating. Signals that require data conversion and calibration include pressure, temperature and flow. The processed data was stored in the PLC data register DB. The PLC data register DB block was capable of power-off hold, which was convenient for researchers to operate. The running process of the CO_2_ transcritical refrigeration experimental platform was designed as shown in Fig. 5.

### HMI system design

**Figure 6:**
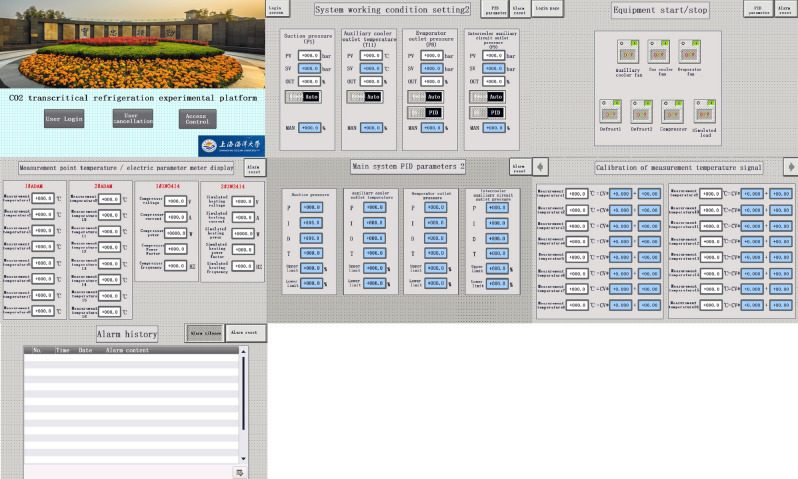
Touch screen page.

The operating status of the EXP can be displayed by the HMI, and researchers can control the experimental equipment in the EXP through the HMI^27^. The EXP used a SIMATIC 9-inch KTP900 basic touch screen, which data interaction with the CPU through Ethernet communication. The touch screen interface was designed by using the TIA Portal V16 configuration software. The addresses of the input and output signals in the CPU were stored in the data register DB block. The I/O fields and buttons in the touch screen can invoke the data in the data register DB block. Therefore, the data in the data register DB block can be displayed and changed by the touch screen. To ensure data accuracy, the type of data displayed in the touch screen was Real. As shown in Fig. 6, the touch screen includes seven types of operation pages, which were login page, alarm information page, signal calibration page, signal display page, equipment start/stop page, control mode switching page, and PID parameter adjustment page. Set user access rights by configuring user management. To prevent misoperation by researchers, access the operation page by entering the correct password. The discrete variable alarms and alarm views were configured. The discrete variables used for alarms come from the PLC data register M. The alarm information page can display the detailed information of the alarm in real-time, and the alarm can be reset in this page.

**Figure 7:**
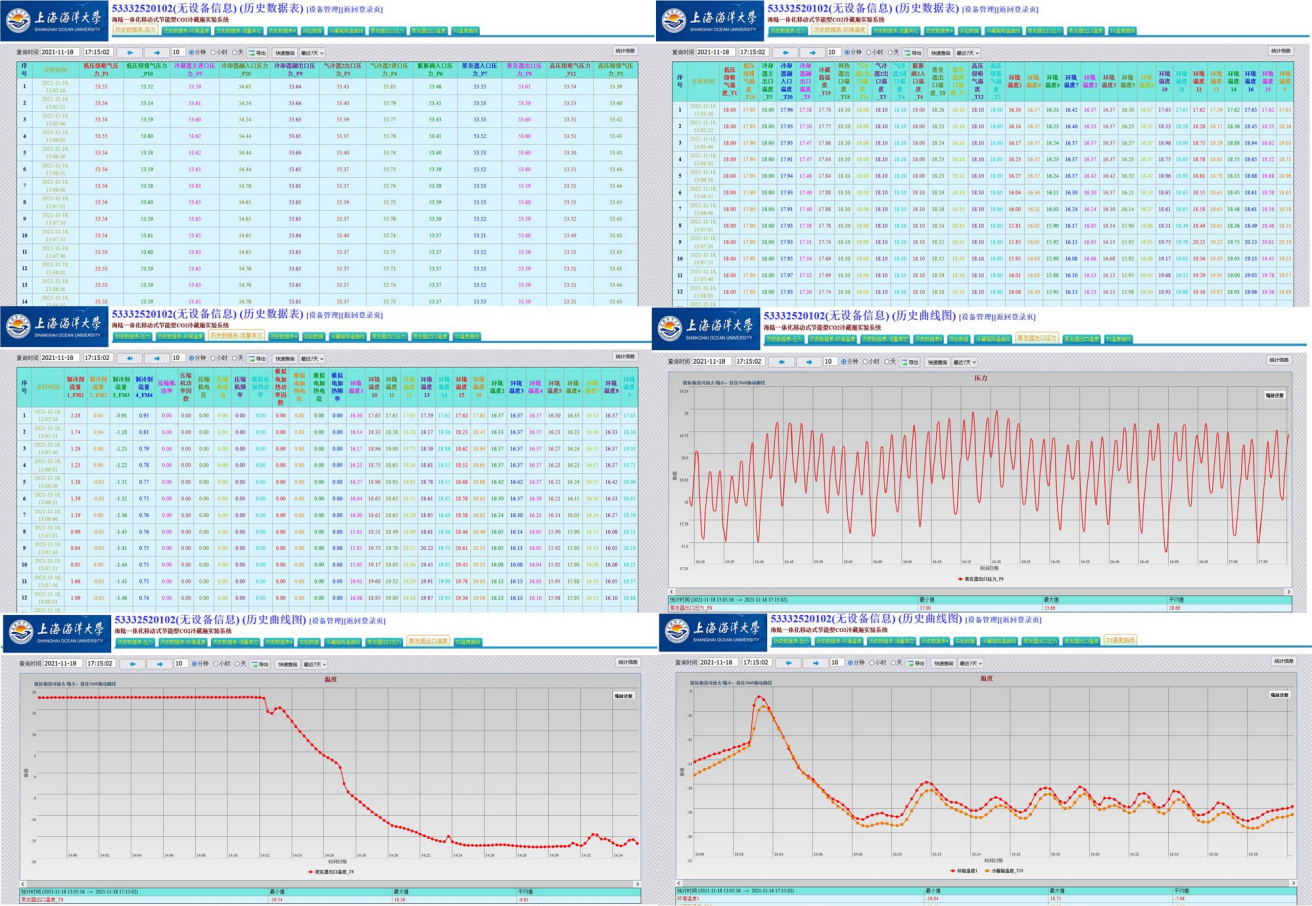
Remote web monitoring page.

IOT module adopts Giant Control GRM533YW-C, which supports wired, 4G and WIFI hotspot internet access. GRM533YW-C can meet the required number of I/O points used for communication in the EXP. The IoT module has two LAN interfaces and one WAN interface. Through the LAN interface, the IOT module was in the same LAN with PLC and touch screen to realize data interaction. Both the remote device and the touch screen can be used independently for the control and data acquisition of the experimental rig. The researcher can download PLC and touch screen programs on the remote device through the IOT module connected to the network. The remote web monitoring page is shown in Fig. 7. The remote web monitoring page can start and stop the equipment, switch the control strategy of the experimental equipment and automatic real-time collection of equipment operation data. In the remote device, the experimental data can be displayed in the form of graphs and can be downloaded, so that researchers can further process and analyze the experimental data.

### PLC communication with LabVIEW

**Figure 8:**
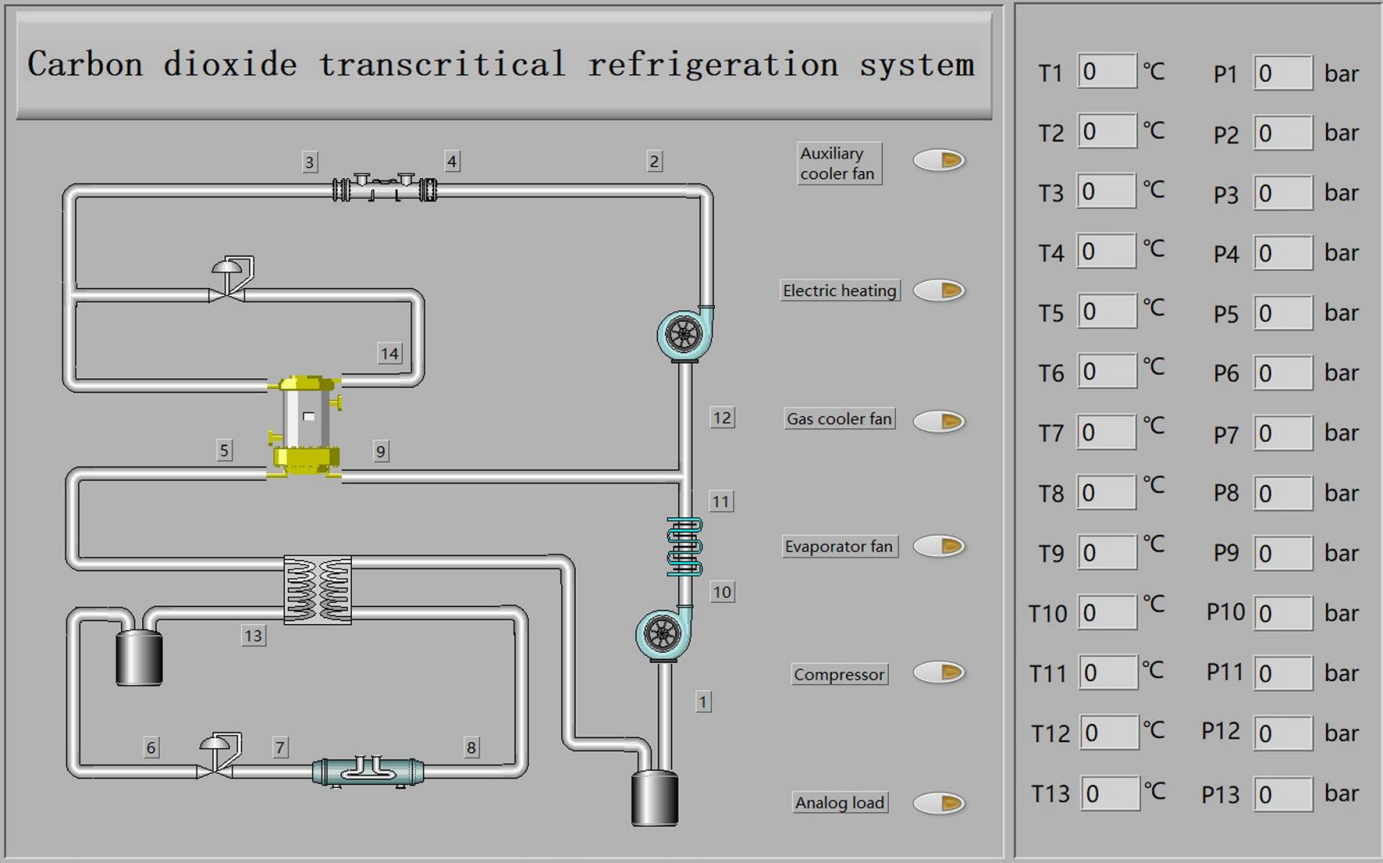
LabVIEW operation page.

**Figure 9:**
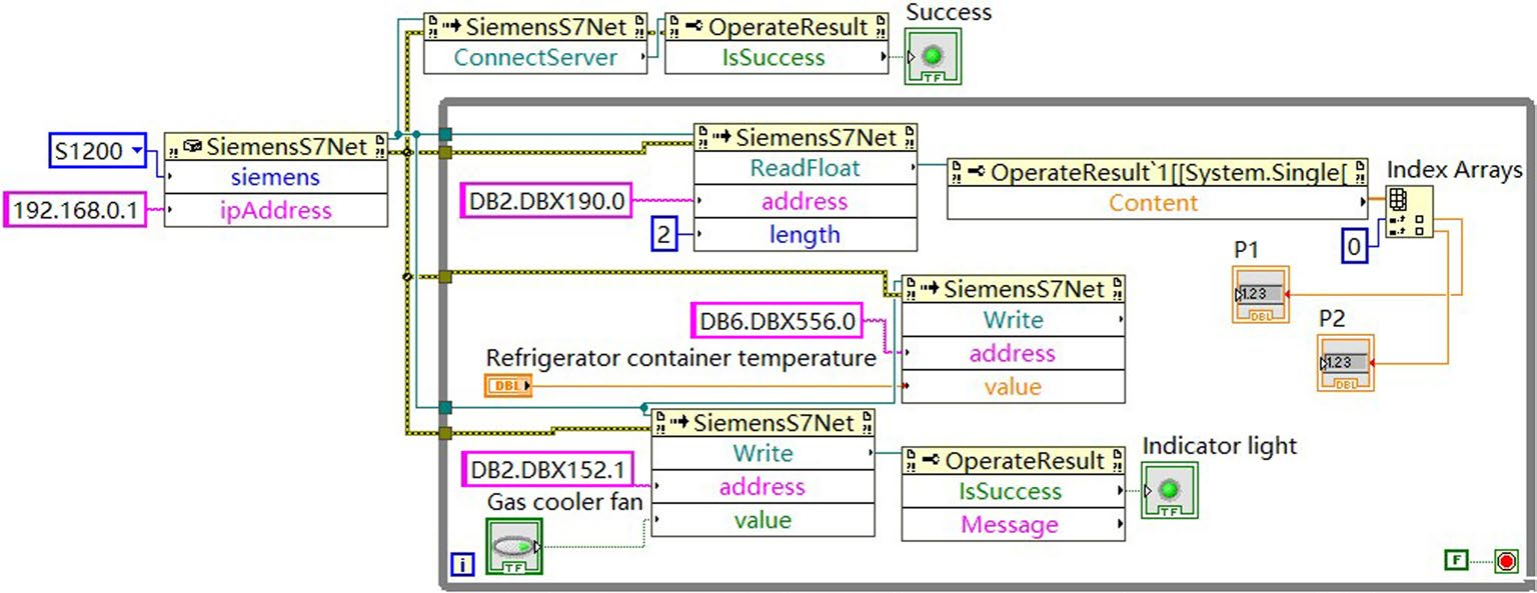
Part of the communication program in LabVIEW.

The LabVIEW as control center was applied to the CO_2_ transcritical refrigeration experimental platform. The LabVIEW operation page is shown in Fig. 8. LabVIEW has a large function library and is programmed graphically. LabVIEW implements more complex industrial control compared to PLC. The PLC and PC were connected through LAN interface for the communication of LabVIEW and PLC. The PLC communication parameters were set to allow PUT/GET communication access from remote objects by TIA Portal V16 software. In the LabVIEW, HslCommunication.dll was invoked and the PLC model and IP address were set. The LabVIEW communication with PLC S7-1200 was realized by reading and writing to the PLC data registers. Part of the communication program in LabVIEW is shown in Fig. 9. LabVIEW can receive signals from the PLC and send signals to the PLC. Make LabVIEW as the master computer and PLC as the slave computer. The CO_2_ transcritical refrigeration system can be controlled through LabVIEW.

## CO_2_ transcritical refrigeration experimental platform control strategy

The control strategy has a significant impact on the stability and accuracy of the refrigeration system operation. In the refrigeration system, the compressor and the EEV are important components. The compressor is able to regulate the cooling capacity in the refrigeration cycle^28^. The opening of the EEV is closely related to the superheat of the refrigerant at the outlet of the evaporator. The degree of superheat has an impact on the stability of the refrigeration system operation^29,30^. To improve refrigeration performance, the control study of compressor and EEV is necessary.

### Compressor speed control

**Figure 10:**
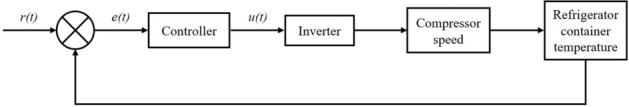
Compressor speed PID controller control schematic diagram.

To achieve good control effect and energy saving of the refrigeration system, optimizing the compressor speed control is the common way. The temperature in the refrigeration container can be controlled by the compressor speed control. The speed of the compressor was controlled by the inverter. Output the 0–10 v analog signal by PLC to the inverter which corresponds to 0–100 rps of the compressor speed respectively. The compressor speed was adjusted by PID controller or manually. The above two compressor speed regulation methods were implemented by programming in the CPU. Compressor speed PID controller control schematic diagram is shown in Fig. 10, PID algorithm is shown in Formula(1):

1 $$u\left(t\right)={K}_{p}e\left(t\right)+{K}_{i}{\int }_{0}^{t}e\left(t\right)dt+{K}_{D}\frac{de\left(t\right)}{dt}.$$

where $u\left(t\right)$ and $e\left(t\right)$ are the output signal and error signal. Where ${K}_{p}$, ${K}_{i}$ and ${K}_{D}$ are the proportional coefficient, integral coefficient and differential coefficient.

**Figure 11:**
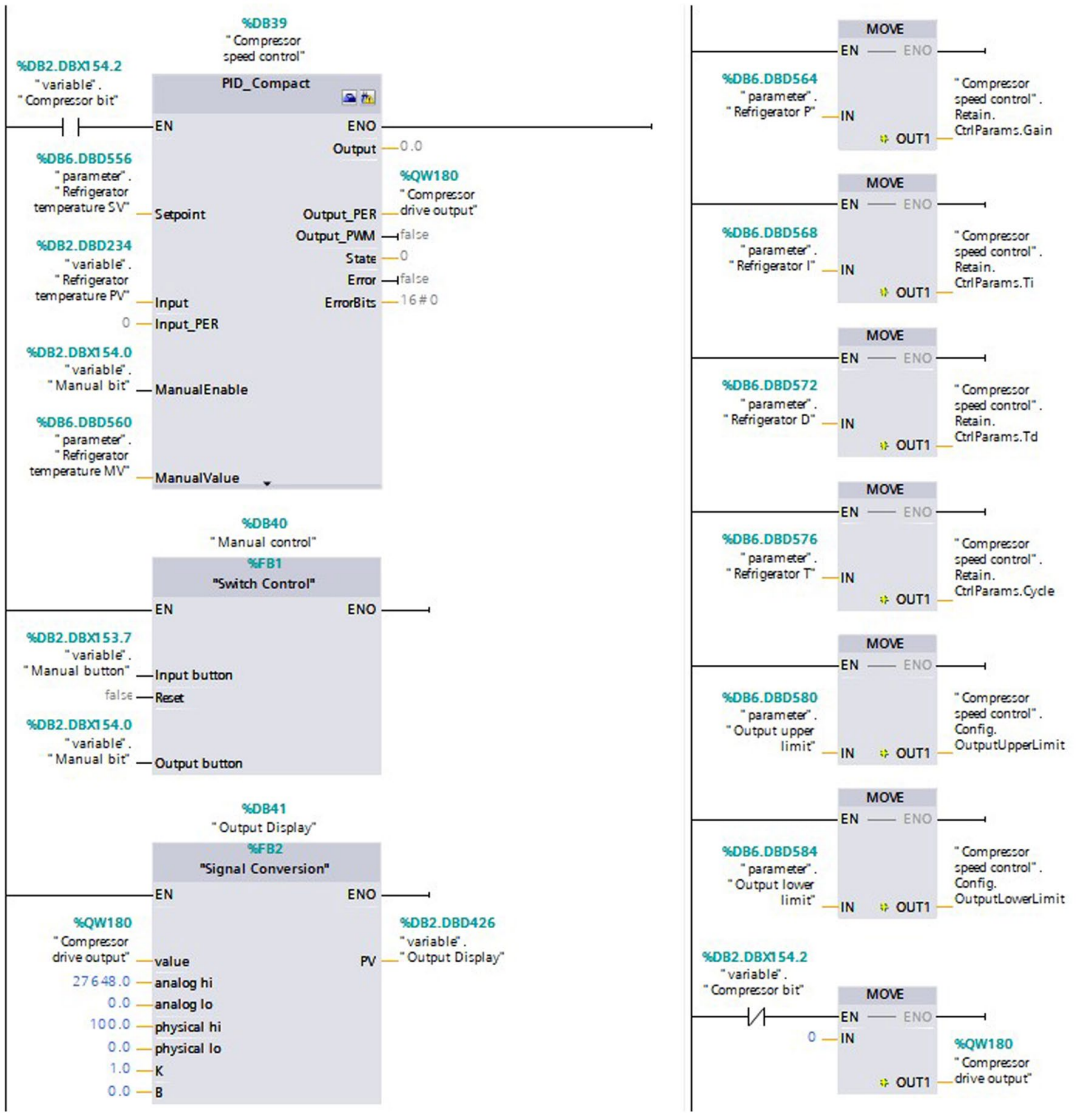
Compressor speed control program.

The PID controller for the compressor speed control program was configured by TIA Portal V16. The controller type was selected as Temperature and the temperature unit was set to °C. Input was selected as PLC Internal Variable and Output was selected as Output_PER. The upper limit of the process value remains default and the lower limit of the process value was set to −50 °C. Manual input PID parameters were opened in the advanced settings and PID controller rules were selected. The PID controller can be invoked by the OB block after completing the PID controller configuration. The PID OB block was a cyclic interrupt organization block which needs attention. The PID controller output was calculated according to the deviation between the set value and the acquisition value. The PID controller adjusts the size of the output analog signal to control the compressor speed. In the PLC program, the manual compressor adjustment method was set. In the manual adjustment method, the manual input value corresponds to the 0–10 V analog signal sent to the inverter. The compressor speed was adjusted by manual input value, which correspond to the percentage of the compressor speed. The addresses of PID parameter adjustment bits and manual input bits were stored in the DB block, so that the HMI program can access and modify them. The PID parameters can be adjusted in real time. Compressor speed control program is shown in Fig. 11.

### EEV open control

**Figure 12:**
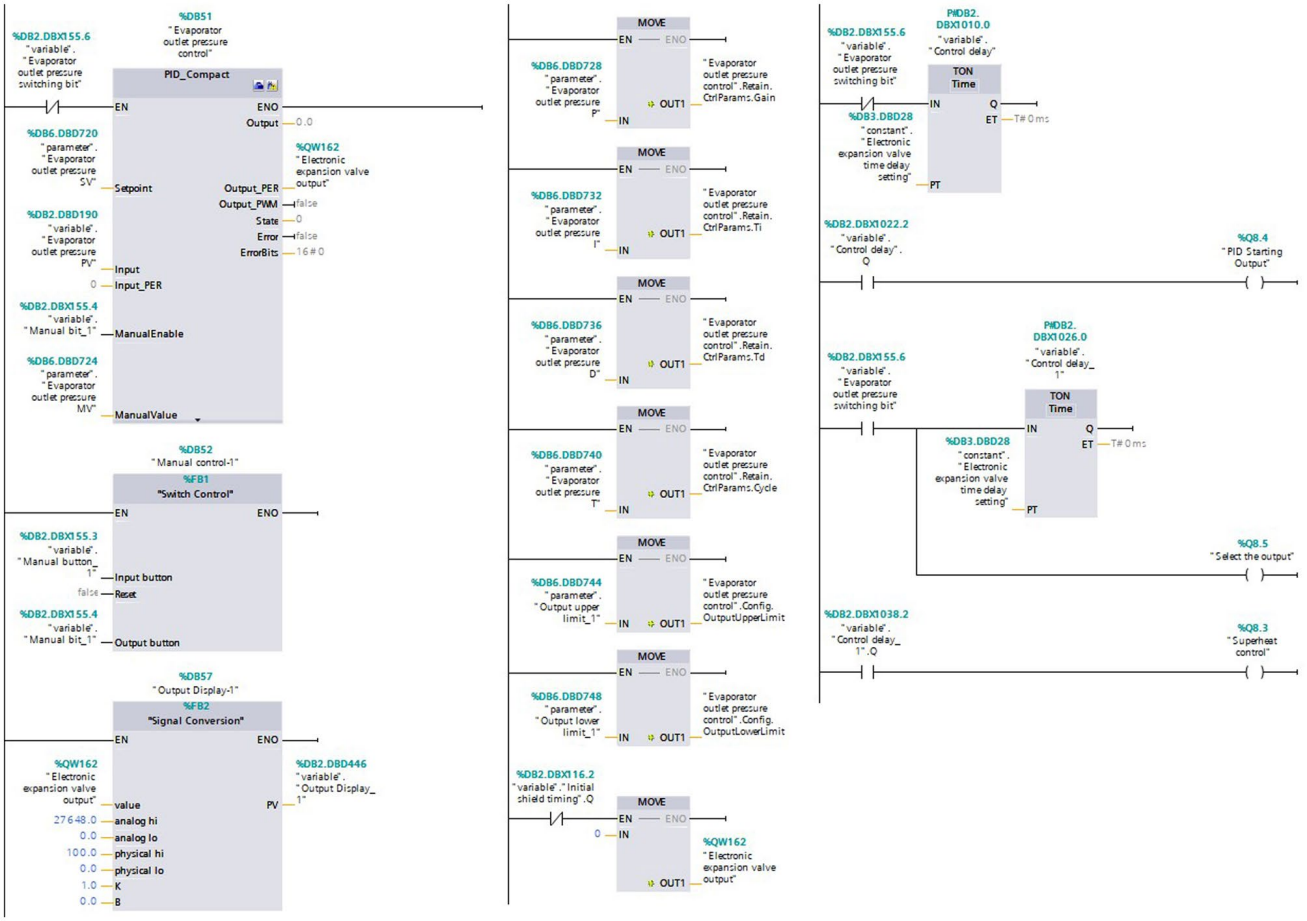
Main circuit EEV open degree control program.

To ensure the stable operation of the refrigeration system and to meet the research requirements, two control methods were designed for the main circuit EEV opening: one was the evaporator outlet refrigerant superheat controller regulation; the other was the evaporator outlet refrigerant pressure PID controller regulation. The experiment rig was installed with the superheat controller (EKE 1C) and the superheat parameters could be set by the connected hand controller (MMIGRS2). The superheat controller can adjust the degree of the main circuit EEV opening in real-time according to the set superheat value during operation. The main circuit EEV opening can be controlled by the superheat controller and the pressure PID controller, which were implemented by programming in TIA Portal V16 software. The PID controller can adjust the degree of valve open to keep the evaporator outlet refrigerant pressure at the set value. The EEV controller (EKE 1A) controls the degree of the valve opening by the 0–10 V DC voltage signal sent by the PLC. Main circuit EEV open degree control program is shown in Fig. 12.

### System abnormal detection

**Figure 13:**
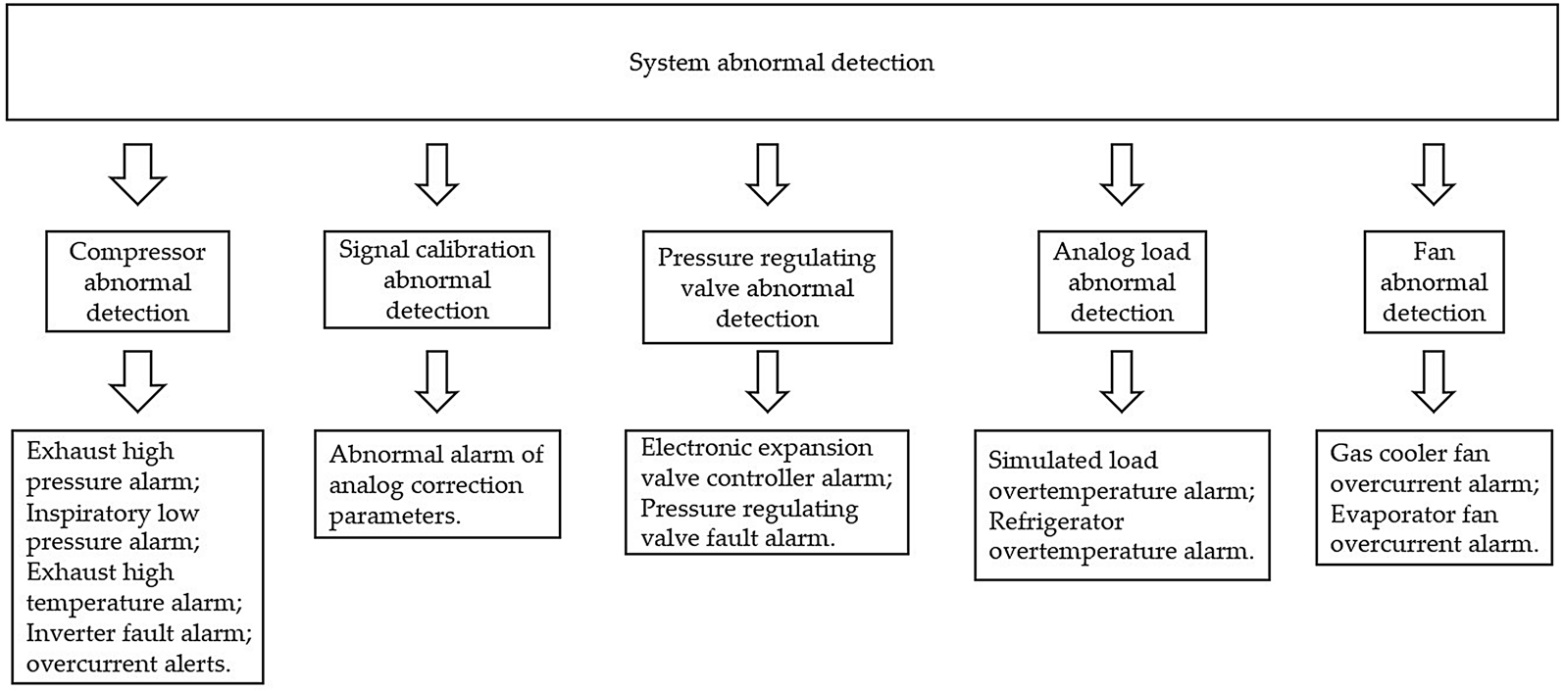
The system abnormal detection schematic.

The maximum pressure of CO_2_ transcritical refrigeration system at operation was about 90 times of atmospheric pressure. The design of abnormal detection program for equipment operation was particularly important^31^. The abnormal data detection program was to prevent accidents caused by equipment failure and misoperation of researchers. The operating status values in the system were obtained by sensors and the system abnormal detection program compares the operating status values with the normal operating status range. The system abnormal detection schematic is shown in Fig. 13. When the device was operating in the normal operating range, the corresponding alarm bit was closed. If the program detects abnormal data, the PLC control alarm bit opens, specific information about the abnormal data was displayed on the HMI and perform a shutdown operation. The researchers reset the alarm bit after confirming the cause of the fault and resolving the fault. The EXP was able to resume normal operation after restarting the experimental setup.

## Experimental results and discussion of CO_2_ transcritical refrigeration experimental platform

The cooling performance of the refrigeration system and the stability test of the control system were carried out. The cooling experiments were performed at ambient temperature of 18 °C. Control operations were performed in the touch screen. The main circuit EEV opening degree was controlled using the superheat controller. The refrigerant superheat at the evaporator outlet was set to 8 K. The speed of the compressor was controlled by LabVIEW as the master computer. The load step experiment was performed after the temperature in the refrigeration container enters the stability zone. The experimental data were downloaded by remote devices and processed by the researchers at the end of the experiment.

### Cooling experiment

**Figure 14:**
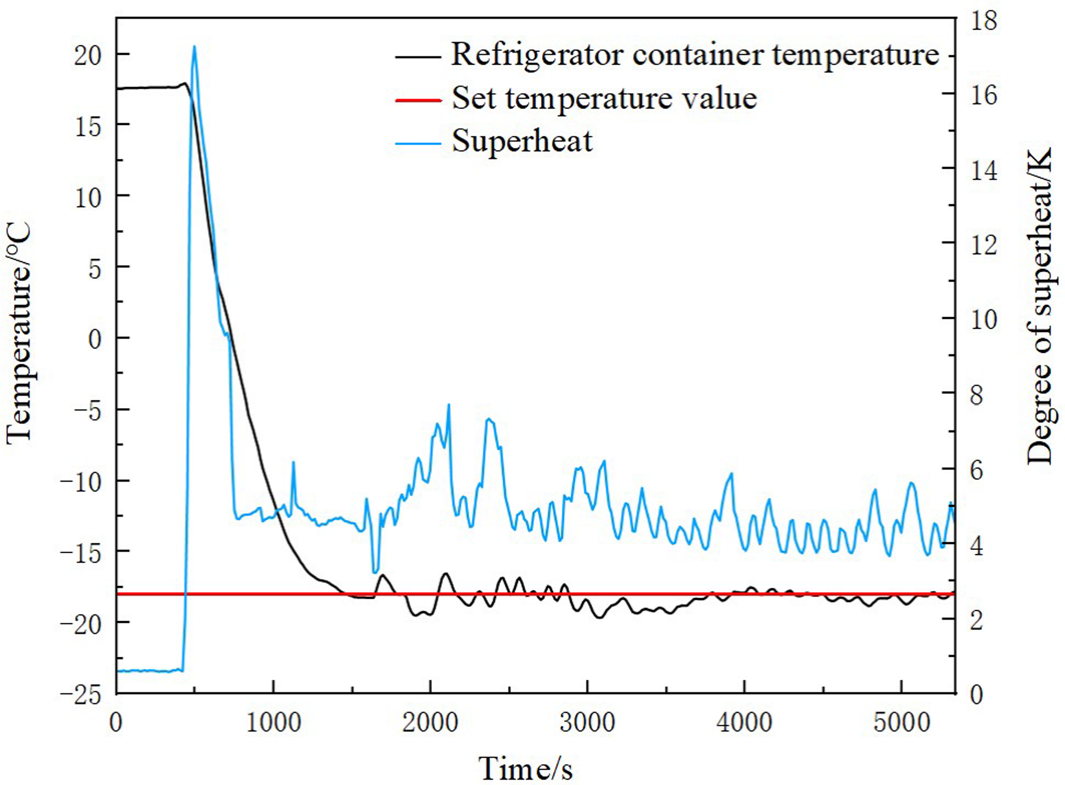
The change of refrigerator container temperature and superheat in the cooling experiment.

The change of refrigerator container temperature and superheat in the cooling experiment is shown in Fig. 14. The refrigerator container temperature was set to −18℃ and add load 600w to simulate the actual goods. As can be seen in the figure, the refrigeration container temperature decreased from 18 °C to −18 °C in 1035 s, with average temperature decrease of about 2.09 °C per minute. The maximum deviation value of the temperature in the refrigeration container after stabilization was less than 0.4 °C. At the beginning of the cooling experiment, the superheat rises significantly. This was caused by the rapid reduction of the evaporator outlet pressure due to the initial start of the compressor. The superheat stabilizes after about 300 s of starting the compressor. The superheat was not maintained at the set superheat value when the temperature in the refrigeration container was stable. This phenomenon was caused by the analog load was small and the compressor speed was low. The evaporator outlet pressure was relatively high, and the superheat degree exceeds the range that can be adjusted by the main circuit EEV. It makes the superheat stabilize at a lower value. This can be solved by increasing the compressor speed or increasing the load. In the cooling process, the refrigeration container cooling down quickly and the refrigeration system runs smoothly. And when the temperature decreases to the set value, the degree of superheat and refrigeration container temperature fluctuations were small. This indicates that the EXP control system was well combined with the refrigeration system, and the platform control was stable.

### Load step experiment

**Figure 15:**
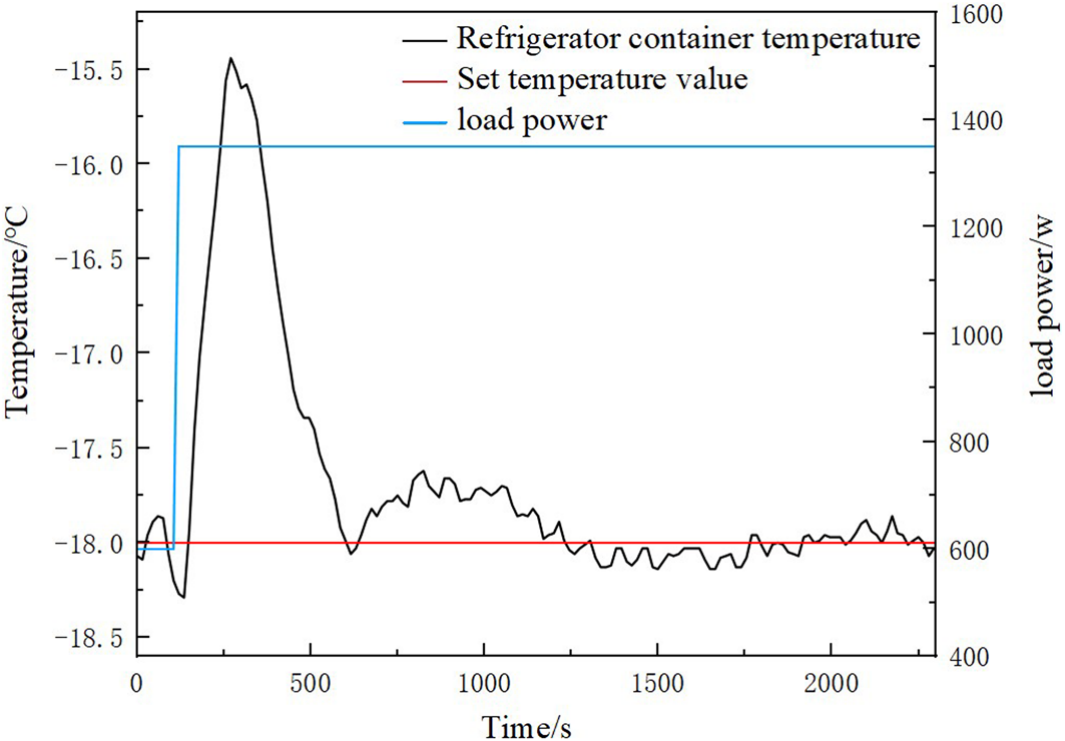
The temperature change process of the refrigeration container in the 750 W load step experiment.

**Figure 16:**
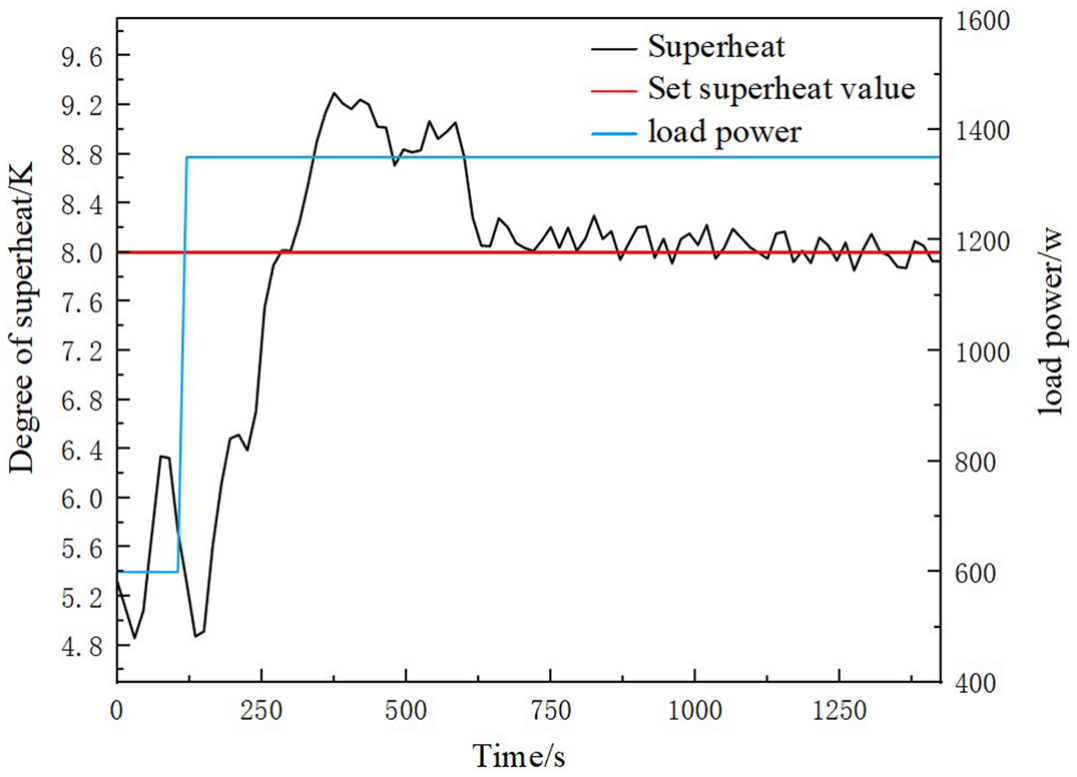
The superheat change process in the 750 W load step experiment.

An additional 750 W load step was added to the system when the refrigerator container temperature was stabilized at −18 °C. Figures 15 and 16 show the temperature change process of the refrigeration container and superheat change process in the 750 W load step experiment, respectively. As can be seen in Fig. 15, after adding the load step, the previous dynamic balance was disrupted. The refrigeration container temperature rises rapidly to its maximum value, reaching a maximum error of 2.56 °C. After that, the compressor speed was adjusted through the LabVIEW. The set temperature was reached again after 450 s, and the static error of the refrigerator container temperature was ± 0.2 °C. As can be seen in Fig. 16, the superheat of the refrigerant at the evaporator outlet rises significantly after adding the load step, reaching a maximum error of 1.3 K. This phenomenon was caused by the increase of the refrigerant temperature at the evaporator outlet due to the increased load in the refrigeration container. After that, as the speed of the compressor and the main circuit EEV opening degree were combined to adjust, the superheat decreases and stabilizes. The transient time of superheat was 460 s, and the static error of the superheat was ± 0.17 K. The static errors of refrigerated container temperature and superheat obtained in this paper are less than ± 0.5 °C and ± 0.2 °C , respectively, obtained from the system studied by Chen et al^32^. In summary, this EXP has effective control of the temperature of the refrigeration container and can quickly respond to load steps. The adjustment ability of load fluctuation was strong, and the temperature fluctuation was small in the case of load fluctuation. The EXP can achieve the requirements for control accuracy and experimental stability.

## Conclusion

PLC and HMI programs were applied in the CO_2_ transcritical refrigeration experimental platform to realize the refrigeration system control, data acquisition and abnormal alarm. The research conducted in this paper was detailed as follows.

1. The PLC communicated with the touch screen and the IoT module through PROFINET, realizing data interaction in the same LAN. The CPU received the analog signals from the sensors through the PLC expansion module. Through the MODBUS RTU protocol, the PLC acquired data from the EPM and ETAM, which further extended the number of acquisition points of the EXP. The conversion and processing of the analog signals was performed in the PLC. Through the touch screen and remote device, the display and storage of experimental data was realized.
2. The compressor speed could be PID controller controlled and manually controlled, and the main circuit EEV could be superheat controller controlled and PID controller controlled, which could be realized by CPU. The analog signals calculated by the CPU were sent to the inverter and the EEV controller to control the compressor speed and the main circuit EEV opening, respectively. The LabVIEW control of CO_2_ transcritical refrigeration system was realized. When abnormal data was detected by the PLC, the EXP will display the detailed information of the abnormal data and perform a shutdown operation.
3. In the cooling experiment, the temperature of the refrigeration container decreased from 18 °C to −18 °C in 1035 s and the maximum deviation value of the temperature was less than 0.4 °C. In the 750 W load step experiment, it took 450 s for the refrigeration container temperature to reach −18 °C again, and the static error of the refrigerator container temperature was ± 0.2 °C. In the 750 W load step experiment, the transient time of superheat was 460 s, and the static error of the superheat was ± 0.17 K. The control system worked well with the refrigeration system, and the control accuracy was high and the system stability is good.

This paper provided a design reference of the control system for the other refrigeration system. In the EXP, the compressor, auxiliary cooler fan, gas cooler fan, EEV and pressure regulation valve in the CO_2_ transcritical refrigeration system can be controlled with different strategies. Based the experiment on this EXP, the control strategies of the CO_2_ transcritical refrigeration system can be optimized in the future.

## Data Availability

The raw/processed data required to reproduce these findings cannot be shared at this time due to technical or time limitations. However, these data will be shared upon request to the corresponding author.
